# Daily Online Testing in Large Classes: Boosting College Performance while Reducing Achievement Gaps

**DOI:** 10.1371/journal.pone.0079774

**Published:** 2013-11-20

**Authors:** James W. Pennebaker, Samuel D. Gosling, Jason D. Ferrell

**Affiliations:** Department of Psychology, University of Texas at Austin, Austin, Texas, United States of America; University of Minho, Portugal

## Abstract

An in-class computer-based system, that included daily online testing, was introduced to two large university classes. We examined subsequent improvements in academic performance and reductions in the achievement gaps between lower- and upper-middle class students in academic performance. Students (N = 901) brought laptop computers to classes and took daily quizzes that provided immediate and personalized feedback. Student performance was compared with the same data for traditional classes taught previously by the same instructors (N = 935). Exam performance was approximately half a letter grade above previous semesters, based on comparisons of identical questions asked from earlier years. Students in the experimental classes performed better in other classes, both in the semester they took the course and in subsequent semester classes. The new system resulted in a 50% reduction in the achievement gap as measured by grades among students of different social classes. These findings suggest that frequent consequential quizzing should be used routinely in large lecture courses to improve performance in class and in other concurrent and subsequent courses.

## Introduction

Recent figures regarding graduation rates at U.S. colleges have sounded widespread alarm about the level of college preparation provided by high schools, especially for students from economically disadvantaged backgrounds [1]–[3]. On entering college, many students lack the basic content knowledge that is needed for the mastery of courses in math, science, and other disciplines [4]. In other cases, students have deficits in procedural knowledge – the how of learning – which some have called self-regulated learning [5]–[6]. This procedural know-how underlies the skills students must develop to acquire content knowledge, including the basic ability to take notes, to study, to monitor their performance, and to think critically in ways that optimally prepare them for exams and other assessments [7].

One challenge colleges and universities face is efficiently training students to learn these basic self-regulatory skills [8]. Not having acquired such skills has been implicated as particularly problematic for students whose families and neighbors have less education and are subject to more social and economic barriers [2], thus accounting for some of the class-based differences in college performance [9]. Is it possible to train new college students–especially those from disadvantaged backgrounds–to learn and perform better in a classroom setting?

One important self-regulatory method to improve preparation and performance is to give students frequent testing along with rapid, targeted, and structured feedback on their performance [2], [10]–[11], so that they can adjust their learning and studying strategies in time to improve their performance in a course [12]–[15]. Recent research has demonstrated that the mere act of testing helps students to remember and retrieve information more efficiently [16]–[19]. Indeed, studies relying on mastery learning principles have found that frequent testing results in substantially improved performance [15]. In other words, repeated testing of students does much more than assess learning skills: it is a powerful vehicle that directly enhances learning and thinking skills [20].

In light of the benefits of frequent testing with immediate feedback, colleges might benefit from adopting these methods during students’ first semesters so they can continue to benefit from the learning skills. However, frequent testing is difficult to implement at scale using traditional teaching methods because of the prohibitive amount of effort required to write and grade exams in anything other than very small classes [21].

The current revolution in computer technology is ushering in new methods by which it is possible to teach hundreds, even thousands, of students and to deliver frequent exams that include immediate and personalized feedback. Here we introduce TOWER (Texas Online World of Educational Research), a new scalable online teaching and learning platform that provides students with recurring and immediate feedback on their performance as they learn material. The goal of this project is to compare the performance of students taught using TOWER with students taught in previous years with traditional teaching and testing methods. Specifically, we tested whether the TOWER-based method of repeated testing would result in improvements in current and subsequent course performance and, at the same time, reductions in the well-known performance disparities across social class.

## Methods

### Participants

The participants were all students who registered for two large Introductory Psychology courses in the Fall 2011 semester (the “TOWER group”; N = 982) or two virtually identical classes in the traditionally taught “Comparison group” (N = 993) in Fall 2008. As can be seen in Table S1, the final sample size of the classes were 901 for the TOWER group and 935 for the Comparison group, reflecting a slightly higher withdrawal rate for the TOWER class than the Comparison class (11.9% versus 9.6%). As shown in Table S1, the demographic data for the two courses were comparable in terms of sex ratios, year in school, racial and ethnic composition, and mean level of parental education. Note that for analyses based on class grades in the semester following the Introductory Psychology classes, data from 5.6 percent of students from both classes were lost due to their not registering for courses in the Spring. The final sample for the full Spring analyses was 1,732 (861 for TOWER and 871 for Comparison classes).

### Context

The two first authors jointly taught two back-to-back large Introductory Psychology classes together each Fall semester from 2006–2011, excluding 2009. For all classes, both instructors stood together in front of large classes and contributed equally. Classes from 2006–2010 were conventionally assessed with four class-long exams over the semester, which accounted for approximately 86% of the final grade (four writing assignments accounted for the remaining 14%). The in-class exams typically included 40–45 multiple-choice questions that were machine-graded using Scantron forms. In addition, students relied on a standard textbook for the daily reading assignments.

The TOWER class had the same lecture format as previous years. The primary difference was that students were required to bring wifi-enabled devices to every class so that they could connect to TOWER. The first 10-minutes of each class were devoted to an 8-item daily quiz. Seven of the questions covered material from the previous lecture and readings. The remaining item was a personalized question consisting of a question the student had answered incorrectly on a previous quiz. In the unlikely event that the student answered all previous questions correctly, TOWER randomly selected another question that he/she had taken earlier in the semester. The final grade was based on quizzes (86% of the total grade) and four writing assignments (14% of the final grade – see Text S1 for more detail). A second substantive difference between the TOWER class and earlier years was that no textbook was assigned. Rather, all readings came from online sources. The only reason that 2008 was used as the Comparison class was because it was the only recent class that employed the same demographic survey.

### Intervention

The TOWER online platform was developed to deliver daily in-class computerized quizzes. Students took 26 8-item multiple-choice quizzes at the beginning of every class via their own laptops, tablets, or smartphones. No final or other exams were administered. Across both years, the large classes were held twice a week and there were no discussion groups. Both courses employed approximately one teaching assistant for every 200 students.

Over the course of the 2011 semester, classes met 28 times. There was no quiz on the first day of class and, to allow students to familiarize themselves with the quiz procedures, the scores from the first quiz (held during the second class) did not count towards the class grade. Overall, then, 26 quizzes contributed to the final course grade. Students dropped their three lowest quizzes. In addition, they could take up to five quizzes remotely (e.g., in their dorm). If six or more were taken remotely, the students had to take a comprehensive final exam to substitute for those after the first five; 15 students took this option to replace one or more quizzes.

Approximately a third of the test questions came exclusively from lectures, a third from the readings, and a third from a combination of the two. In general, the readings were intended to complement the lectures rather than overlap with them.

### Ethics

This project was recognized as having exempt status (under 45 CFR 46.101(b)(4)) by the University of Texas at Austin Institutional Review Board (reference number 2012-07-0064) on July 23, 2012. That is, this research is considered exempt from review and the need for written informed consent because it is considered educational research and the data were analyzed with all identifying information removed after the class was concluded. Students in both the TOWER and comparison groups were informed on the first day of class that all measures of academic performance, surveys given in class, and other information provided by the University would be analyzed at the conclusion of the class in anonymized format.

### Measures

#### Socioeconomic status (SES) and parental education

Socioeconomic status, or SES, was measured by parental education, a widely used indicator of SES in education research [22]–[23]. As our SES proxy, students were asked to rate separately their mother’s and father’s highest level of education along a 7-point scale: no high school, some high school, high school graduate, some college, college graduate, some graduate school, professional degree. The mean of the parents’ education was computed. If information was only available for one parent, only that parent’s data was used.

#### Class performance

Student performance was evaluated in several ways. The first involved the analysis of students’ overall grade based on quizzes and writing assignments. Grades in all classes at the university are assigned ranging from the highest grade of A to a failure grade of F. The letter grades are routinely converted to a grade point average, or GPA, index where A = 4, B = 3… F = 0. GPAs can be directly compared across different courses and semesters.

The second approach directly compared the quiz performance of the TOWER class with the performance of students who had taken the course in previous years. Beginning on the ninth quiz, one question was selected from a test given in an earlier course taught by the same instructors. The teaching assistants chose the previously used test items and determined if they were relevant for the day’s quiz only after the lecture had been delivered. Thus, the instructors were blind to the test items on the day of the lecture so they could not have lectured in way that favored those questions. The test bank was publically available for 2011 and all previous classes on the class website. For the 2011 TOWER class, the test bank went back eight years and was based on 44 separate exams, each with 40–45 questions (i.e., approximately 1,900 questions in total). Students had access to all of the old tests but they were organized in ways that did not match the quiz system and were based on more traditional textbook-influenced courses. This analysis strategy was only possible for the TOWER class because there was no comparable benchmarking strategies in previous years.

The University Registrar Office provided GPAs for all students enrolled in the TOWER and comparison classes for the other courses they were taking concurrently with their introductory psychology class as well as in the semester following their introductory psychology class. In addition, the Registrar also provided number of courses (in the form of semester credit hours) taken in each for each semester and College Entrance Exam Board scores (standardized equivalence of the Scholastic Assessment Test, or SAT).

#### Attendance and other data

Attendance was not required nor directly collected so was estimated by measuring the number of students who completed in-class surveys via Scantrons (in the Comparison class) and TOWER. These surveys were administered in most classes. Although filling out the surveys was voluntary and had no influence on students’ grades, most students in attendance completed them. The only motivation to complete the questionnaires was to learn about the surveys themselves and to get personalized feedback about their responses. In theory, students not attending class could complete the surveys on TOWER, but they would not know when to access them because surveys appeared on TOWER only for a narrow window after being introduced by the instructors during lecture and before moving on to the next topic.

Finally, the university required standardized course instructor surveys during the last two weeks of classes. Although the surveys were anonymous, the means and standard deviations for each class were made available once the course was finished.

## Results

### Class Grades and Performance

#### Average grades and test performance

On the surface, students in the TOWER-based class made slightly lower grades compared with students in previous years. Of the 901 students who did not drop the course or did not take the course pass/fail, their final course grades were A (14.4%), B (39.3%), C (29.1%), D (10.0%), F (5.9%). The mean TOWER GPA for those receiving a letter grade was 2.47 (*SD* = 1.05) which was significantly lower than the Comparison class grade of 2.59 (*SD* = 1.03), *t*(1832) = 2.43, *p* = .02 (two-tailed test), Cohen’s *d* = .11. Identical effects emerged using a mixed model regression including parental education and year of course showing an overall higher psychology grade point in the Comparison class than the TOWER class (β = −.06, *t* = 2.50, *p* = .01, *d* = .12).

Unfortunately, these findings are misleading because the Comparison class was artificially inflated (or curved upwards) by the instructors but the TOWER class was not. Specifically, in years prior to the TOWER class, the first of the four exams was curved upwards by adding the equivalent of 0.9 letter grade to each student’s grade (from a mean of 63% to 72%). On the second exam, the across-the-board curve was 0.5 letter grade. No curve was added to the TOWER grades because students could drop their three lowest benchmark quizzes. The purpose of the curve was to reduce the number of students who failed the first exams – a standard practice in American universities.

A more direct test of the performance of the TOWER class involved a direct comparison of students’ performance on the same test questions given in previous years. As described earlier, beginning on the ninth quiz in the TOWER group, a single question was included from the tests administered in earlier years. On these 17 benchmarked questions (i.e., administered in quizzes 9–26), the TOWER group performed the equivalent of half a letter grade better compared to students prior to 2011 (77.1% versus 71.2% correct, paired-*t*(16) = 2.01, *p* = .06, two-tailed, *d* = 1.01). These analyses suggest that the actual course grade in the TOWER class (which, unlike the Comparison class, had not benefited from upward curving) underestimated performance by 0.59 of a letter grade.

**Figure 1:**
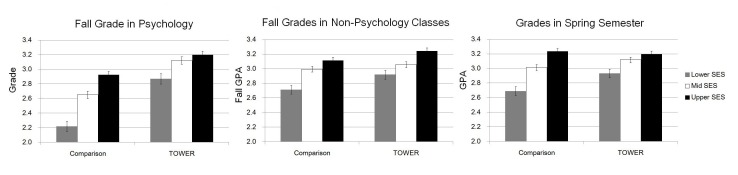
Grades in Fall Psychology and Non-Psychology Classes and Spring Classes by Year and SES. The sample size for these analyses is 1732. Although the 2011 TOWER class was lower (mean GPA = 2.43) than 2008 comparison group (GPA = 2.59), actual performance using a benchmarking procedure revealed that the TOWER students performed 0.59 letter-grades above the comparison students. This adjustment is included in figure.

Because the actual grades did not reflect performance, data for the TOWER class were analyzed in two ways: one based on the actual grades the students received on their quizzes and the second based on their relative performance based on the benchmarked grades. The actual statistical analyses are based on the raw unadjusted GPAs. Figure 1, however, adds a constant of 0.59 to the TOWER group’s psychology grade that reflects students’ actual performance relative to the Comparison class’s performance. Note that adding a constant did not change any of the interactions with SES.

### Class Grades in and Outside of Psychology in the Fall and Spring

If the TOWER system promoted self-regulated learning skills, the skills should generalize to performance in other classes. A 2 (Course: TOWER versus Comparison)×3 (Course-GPAs: Fall psychology class, other Fall classes, all Spring classes) repeated measures ANOVA revealed a main effect for course GPAs, *F*(2, 1756) = 330, *p*<.001, *d* = 1.23; and, more importantly, for the Course by Course-GPA interaction, *F*(2, 1756) = 23.6, *p*<.001, *d* = 0.33. Overall, TOWER students’ grades were higher in their other concurrent classes in the Fall semester (3.07 versus 2.96 for the comparison course) and the subsequent Spring semesters (3.10 versus 2.98).

### SES Disparities in Class Performance

SES was split into three groups based on parental education. The three groups used in these analyses are referred to as upper-middle class (mean parental education = some graduate work), middle class (college graduate), and lower middle-class (some college or less).

#### Performance

A linear regression entering Year, SES level, and the interaction indicated that the GPA differences between SES levels were greater in the Comparison than in the TOWER courses (interaction effect: β = −.05, *t* = 2.25, *p* = .03, *d* = .10). This analysis points to a narrowing of the traditional achievement gap [23]–[25] of students taking Introductory Psychology (see Figure 1). A simple comparison of grade differences between the upper middle class and lower middle class students was significantly smaller in the TOWER class than in the Comparison class (0.34 letter grade difference versus 0.71), in short, a reduction of the achievement gap by over 50%.

#### Class performance in and outside of psychology by SES

Additional analyses of the daily quizzes suggested that lower SES students tended to perform at rates similar to middle class students until the last 2–3 weeks of the class (see Text S1 and Figure S2). Why would this be the case? One possibility is that repeated testing allows students to better estimate their overall course standing. If so, by the last weeks of the class, they should have a fairly good sense of what their final grade would be. So, instead of studying so much for their psychology class, they put additional energy into their other courses. If true, students in the TOWER class should perform better in their other classes compared to the Comparison group.

An overall 2 (Course: TOWER versus Comparison) by 3 (SES: lower middle, middle, upper middle) by 3 (Semester GPA: Fall Psychology class, other Fall classes, Spring semester classes) between-within ANOVA was run. As depicted in Figure 1, there was a significant SES main effect, *F*(2, 1726) = 39.3, p<.001, *d* = 0.48, such that higher SES was associated with higher overall grades. There was a Course by SES interaction, *F*(2, 1726) = 3.18, *p* = .04, *d* = 0.12. There were also effects for Semester GPA, *F*(2, 3452) = 363.5, *p*<.001, *d* = 0.92, and SES by Semester GPA, *F*(2, 3452) = 23.0, *p*<.001, *d* = 0.23. Most impressive, however, was the emergence of the Course by SES by Semester GPA three-way interaction, *F*(4, 3452) = 2.81, *p* = .024, *d* = 0.11. Not only are the TOWER students performing better than the Comparison students but the effects are magnified for the lower-middle and middle SES students, suggesting that benchmark testing is reducing the achievement gap in other classes taken concurrently and subsequently. The reduction in the gap is noteworthy: a 34% reduction for classes taken outside of the psychology courses in the Fall and a 49% drop in all classes taken in the subsequent Spring.

### Attendance and Other Relevant Data

Completion of questionnaires over the course of the semester served as a way of estimating class attendance. During the first five weeks of class in August and September, the percentage completion rate of surveys was 98.5% in the TOWER class and 87.9% for the Comparison class; for October the numbers were 95.6% and 79.7%; for November, the TOWER class survey participation averaged 88.5% compared with the traditional system’s 65.9% (*χ^2^* (1) = 17.01, *p*<.001, *d* = 0.20).

In addition to attending the TOWER class at higher rates than the comparison class, analyses of the course instructor surveys revealed differences in students’ ratings of the amount of work that the course demanded. All students were asked if the course work was insufficient, light, average, high, or excessive. Along a 5-point scale, where 5 =  excessive, the TOWER class was rated more demanding than the Comparison classes (3.25 versus 3.07), *t* (1188) = 4.19, *p*<.001, *d* = 0.24.

## Discussion

The results indicate that the TOWER class evidenced improved performance in the class itself, in the other classes that the students were taking that same semester, and in the classes they took the following Spring semester. The findings cannot be solely attributed to the learning of psychology content because only a small percentage of students were psychology majors who took subsequent psychology classes. This same logic applies to a number of other potential alternative explanations (e.g., that cheating may have been easier in the TOWER group than in the Comparison group) because such factors might account for the performance differences in the Introductory Psychology classes themselves but they are unlikely to be responsible for the improved performance in other and subsequent classes.

In addition, the effects were not due to grade inflation over time. In fact, between 2008 and 2011, mean freshman GPA in the College of Liberal Arts dropped slightly from 2.80 to 2.78. Nor were the effects due to a change in the caliber of students taking the instructors’ psychology classes–mean SAT scores of students in the classes increased slightly from 1186 (Comparison group) to 1199 (TOWER group) but analyses controlling for SAT scores yielded the same main effects and interactions. Finally, there was no evidence that students were taking a lighter load in either semester.

Another set of alternative explanations draw on variants of the Hawthorne, demand, and expectancy effects [26]–[27]. The general expectancy argument is that research participants show improvement simply because they are aware they are in a study, are aware they are in an experimental group, implicitly want to please the researcher, or are affected by the researchers’ expectations. Several factors mitigate against these possibilities. First, every year the instructors taught this course, they made changes to the class so the TOWER class was not unusual in providing novel methods and content. Second, the instructors were not aware of participants’ SES levels until after the class was over. Third, the fact that daily testing might differentially affect participants from different SES levels did not occur to the instructors until the class was over. Fourth, Hawthorne, demand, and expectancy effects would be unlikely to impact performance in the other and subsequent classes.

In our view, the patterns of improved performance across three outcomes (in Introductory Psychology, in other Fall classes, and in subsequent Spring classes) most plausibly reflect changes in students’ self-regulated learning – their ability to study and learn more effectively. However, measuring improvements in self-regulation skills retrospectively is difficult to do so this causal explanation remains to be tested directly.

In addition, it is not possible to disentangle which features of the TOWER class directly influenced the changes in class performance. Unlike the comparison group, the TOWER group brought computers to class, had online readings rather than a textbook, and participated in a different testing method. However, in our estimation, of all the changes made, the daily benchmark testing was the most significant change and was the only feature that required students to change how they studied and prepared for tests. In addition to the effects in improving retention and retrieval [18]–[20], the quizzes simultaneously accomplished several goals likely to promote self-regulated learning [5]–[6]. The quizzes were constant, providing a structure that facilitated goal-setting behaviors and required students to develop planning and time management skills. In particular, students had to adopt reading, note-taking, and study habits that allowed them to keep up with the material. In talking with students, many noted how they had learned to set aside specific times to prepare for each class–something that they did not initially feel they needed to do for other classes. The repeated testing also broke the material into segments that required students to focus their attention on the relevant content and the immediate feedback after each quiz provided students with a constant and objective means with which to engage in productive self-evaluation. The daily quizzes also encouraged students to attend classes at higher rates.

The frequent testing is the most plausible causal candidate to contribute to increased performance both in the TOWER class and, crucially, in the other classes the students were taking. Moreover, the achievement gap between upper-middle and lower-middle class students in the comparison course was virtually identical to that reported in other studies [24]. The fact that taking the TOWER class could reduce the achievement gap in all of the classes the students took during the year they enrolled in Introductory Psychology is noteworthy.

Although promising, the current project reflects only a case study based on two semesters of large classes. The TOWER course included additional innovations including digital readings rather than a traditional textbook and occasional in-class virtual discussions, which may have contributed to the findings. The current technology is relevant for teaching large synchronous courses where students are able to interact with the instructors and other students, and participate in a more traditional classroom setting. Like other technology-based classroom approaches, TOWER is another sign that we are entering a new era in education and digital technology. The findings suggest that new technologies can boost the learning and performance of students who have traditionally underperformed in college. A major challenge for the future will be to ensure that students and educational institutions can provide the necessary technology at affordable prices.

The teaching methodology behind TOWER raises a number of important questions concerning large enrollment introductory classes. The current study suggests that repeated testing with feedback can bring about both short- and long-term performance improvements [20], [28]–[29]. Future studies must explore whether these behavioral changes reflect alterations in self-regulatory habits (e.g., studying, time management) or, more broadly, in the ways students think and solve problems. One approach to answering this question is through the adoption of mobile technology where students’ daily behaviors are tracked through self-reports or automated processing of geolocation or even biological markers. A related question concerns the degree to which the TOWER approach can be expanded to Massive Open Online Classes (MOOCs). For example, the development of synchronous massive online classes (SMOCs) may be able to influence the self-regulatory and/or thinking abilities of vast numbers of students at a fraction of the cost of current educational methods.

We are entering a revolution in computer-based educational methods. As computer-aided courses become larger and more efficiently run, we will be better equipped to statistically tease out the working ingredients of learning and performance.

## Supporting Information

Figure S1 **View of TOWER students with laptops at the beginning of class prior to beginning the daily benchmark quiz (Photo credit: Marsha Miller, University of Texas, Austin).**
(TIF)

Figure S2 **Standardized quizzes over time by parents’ mean educational attainment.** Note that quizzes have been standardized by day. Values are based on 3-quiz rolling averages. SES is based on mean years of parents’ education where Lower = some college or less (N = 183), Middle = college graduates (N = 439), and Higher = at least some post-college graduate training (N = 280).
(TIF)

Table S1 **Enrollment and demographic statistics of participants.**
(DOCX)

Text S1 **Course Procedures in the TOWER and Comparison Classes (scoring and psychometric properties of the quizzes; course content; online in-class discussion feature; addressing concerns about potential cheating); Analyses of course evaluations; Analyses of quiz performance over time.**
(DOCX)
