# Supplementary figures and images for: Daily Online Testing in Large Classes: Boosting College Performance while Reducing Achievement Gaps
Source: PLoS One. 2013 Nov 20;8(11):e79774. doi: 10.1371/journal.pone.0079774 (PMC3835925; doi:10.1371/journal.pone.0079774)

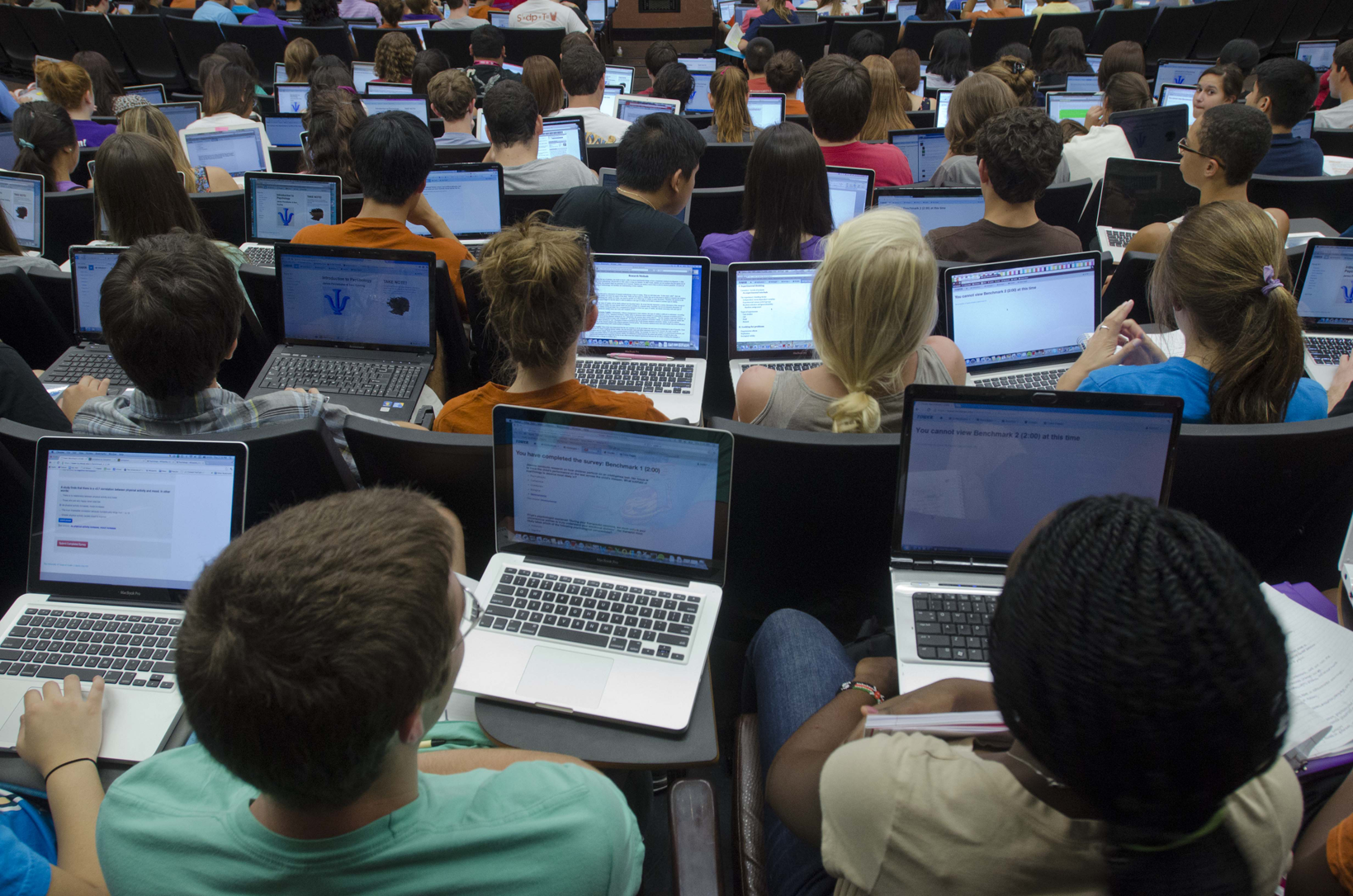

Supplement: Figure S1 — View of TOWER students with laptops at the beginning of class prior to beginning the daily benchmark quiz (Photo credit: Marsha Miller, University of Texas, Austin). (TIF) [file pone.0079774.s001.tif]

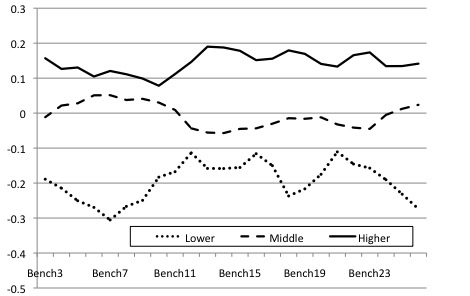

Supplement: Figure S2 — Standardized quizzes over time by parents’ mean educational attainment. Note that quizzes have been standardized by day. Values are based on 3-quiz rolling averages. SES is based on mean years of parents’ education where Lower = some college or less (N = 183), Middle = college graduates (N = 439), and Higher = at least some post-college graduate training (N = 280). (TIF) [file pone.0079774.s002.tif]
