# Supplementary material for: Daily Online Testing in Large Classes: Boosting College Performance while Reducing Achievement Gaps
Source: PLoS One. 2013 Nov 20;8(11):e79774. doi: 10.1371/journal.pone.0079774 (PMC3835925; doi:10.1371/journal.pone.0079774)
Supplement: Table S1 — Enrollment and demographic statistics of participants. (DOCX) [file pone.0079774.s003.docx]

**Table S1**. Enrollment and demographic statistics of participants

|  |  | TOWER (2011) class | Comparison (2008) class |
| --- | --- | --- | --- |
| Enrollment information | Day 1 | 1023 | 1034 |
|  | Drop day (12^th^ day of semester) | 982 | 993 |
|  | Withdrawal from school | 8 | 13 |
|  | Dropped the class | 60 | 26 |
|  | Credit/No credit student | 12 | 21 |
|  | Total students in study | 901 | 935 |
| Demographics (%) | Females | 61.7 | 60.3 |
|  | Year in school 1^st^ or 2^nd^ year | 84.2 | 86.7 |
|  |  |  |  |
| Ethnicity | African American | 4.4 | 5.8 |
|  | Asian American | 16.4 | 15.5 |
|  | Latino/a | 22.0 | 22.2 |
|  | White, not Latino/a | 51.6 | 52.3 |
|  | Other | 5.6 | 4.2 |
|  |  |  |  |
| Mean parental education (%) | Some college or less | 20.4 | 21.7 |
|  | College degree (4 years) | 48.5 | 46.7 |
|  | Post graduate work | 31.0 | 31.6 |
