# Supplementary material for: Daily Online Testing in Large Classes: Boosting College Performance while Reducing Achievement Gaps
Source: PLoS One. 2013 Nov 20;8(11):e79774. doi: 10.1371/journal.pone.0079774 (PMC3835925; doi:10.1371/journal.pone.0079774)
Supplement: Text S1 — Course Procedures in the TOWER and Comparison Classes (scoring and psychometric properties of the quizzes; course content; online in-class discussion feature; addressing concerns about potential cheating); Analyses of course evaluations; Analyses of quiz performance over time. (DOCX) [file pone.0079774.s004.docx]

**Supporting Text S1**

**Course Procedures in the TOWER and Comparison Classes**

***Scoring and psychometric properties of quizzes***. All quizzes included 8 items, 7 of which were from the most recent lecture or readings. Quiz questions were vetted by the authors and a group of six teaching assistants. The primary criterion in writing the questions was that they were conceptually complex and integrated information from multiple sources and/or required students to apply a concept to a novel situation or to explain a phenomenon by using one of several theories or principles. Very few questions were included that relied on rote memory.

The scoring of the quizzes was similar for the Comparison and TOWER classes. Grades on all quizzes were scored based on percent of questions answered correctly, ranging from 0% to 100%. There was no extra credit or bonus questions. Those taking the course Pass/Fail (accounting for 1.2% of total students) received a pass if they averaged at least a 60%, or a D, or higher. 86% of the final course grade was based on test performance and the remaining 14% depended on students turning in four writing assignments. Overall, 80.2% of TOWER and 82.1% of Comparison students completed all four writing assignments.

The psychometrics of the exams were solid. For the comparison classes, students were given four 40-item exams. There were multiple versions of the exams that were not easily identifiable by students. The internal consistency of the exams averaged approximately .75 (with a mean within-test item correlation of .10) and the inter-correlation among the four exams was .59, resulting in an overall alpha of .85. For the 8-item TOWER exams, the average internal consistency was approximately .45 (with a comparable mean within-test item correlation of .10). Across the 27 quizzes, the mean inter-test correlation was .43, resulting in an overall alpha of .95.

One concern about using old test questions to benchmark TOWER performance was that students may have closely studied the old exams which may have resulted in their performing better on the specific benchmarked items. If so, the students would presumably have performed increasingly better on quiz items from old tests as the semester went forward. To test for this possibility, the difference between the percentage correct for the original administrations with the TOWER class administrations was correlated with quiz number. The correlation was non-significant, *r*(16) = -.12, *p* = .64. In fact, direction of the non-significant relationship is opposite to that expected based on this potential confounding explanation. There was no trend to suggest that students performed better on the questions from previous exams as the semester progressed.

***Course content***. Course content was delivered through lectures and reading. The basic lecture information was similar between the TOWER and Comparison classes. The primary difference in non-lecture content was in the nature of the readings. For the comparison classes, students were assigned a popular introductory psychology text, *Exploring Psychology (7^th^ Edition)* by David G. Myers (Worth Publishers, 2008). In the TOWER classes, all readings were from reputable online sources. Note that the assignments included professional articles, information from personal, commercial, and nonprofit webpages, TED talks, and YouTube videos.

***In-class discussions***. In the traditional classes, students were occasionally broken into small groups in order to discuss class topics. In the TOWER classes, an online chat feature was integrated into the TOWER software. On selected days, students were put into randomly assigned chat rooms of 3-7 students where they interacted with one another about class topics. For a discussion of some of the features of the online chats, see Tausczik and Pennebaker [1].

***Cheating concerns***. One possibility is that there may have been differences in cheating in the Comparison versus the TOWER class, which may have differentially influenced the grades. In both classes, a number of safeguards were taken to reduce instances of cheating. Multiple versions of each test were created. In the TOWER class, students could only answer one question at a time and quiz items and the answers were randomized. In both classes, proctors moved about continuously during the quizzes. In both classes, a handful of cheating episodes were detected. After the TOWER class was concluded, an efficient cheating algorithm was built allowing for the instructors to go back to see if patterns of cheating could be detected statistically. Using this method on a subsequent TOWER class resulted in only two instances of repeated cheating during the semester.

It should be noted that the Comparison and TOWER classes were taught in the same auditorium that was designed in such a way that observers standing in the middle and back rows could easily observe groups of at least 80-100 students (See Figure S1). For Scantron and TOWER exams, 5-7 proctors (including the instructors) walked around the room the entire time.

**Course Evaluations**

In both the Comparison and TOWER classes, the same standardized evaluations were given during the last 1-2 weeks of class. In the Comparison class, a mixture of online and in-class Scantron-based evaluations were used. For the TOWER class, only the online course evaluations were employed. It should be noted that the university’s migration to online course evaluations resulted in 516 TOWER students completing the evaluations compared to 760 students in the Comparison class. Course evaluations are not identified by student and are only available in the aggregate.

The overall course instructor survey ratings were slightly lower in the TOWER classes than in the Comparison classes. Along a 5-point scale where 5 = excellent, the overall course rating for the TOWER class was 4.1 compared with 4.4 in the comparison course, *t*(1188) = 5.28, *p* < .001, *d* = .31. One factor that may have influenced students’ lower course evaluations in the TOWER classes was the massive technical hurdles of getting the TOWER system to work at the beginning of the semester. During some of the early classes, entire teams of 8-10 engineers and other IT personnel scurried around the classroom trying to get the system to work efficiently. Similarly, certain computer configurations that included browser applications, spam filters, or other previously installed software programs occasionally prevented students from logging onto the wireless network or onto TOWER. These problems are reflected by the fact that 43% of the students reported having connection problems at least sometimes.

**Tracking Daily Quizzes Over Time**

An alternative way to understand the changing nature of students’ performance over time was to graph and analyze their performance quiz-by-quiz as a function SES. The supporting figure (Figure S2) depicts a 3-quiz rolling average of students’ standardized quiz scores. Consistent with the prediction of differential quiz performance, both main effects and the interaction attained significance: SES main effect *F*(2, 899) = 10.30, *p* < .001, *d* = 0.30; Quiz main effect *F*(23, 877) = 126.10, *p* < .001, *d* = 3.64; SES x Quiz interaction, *F*(46, 1756) = 1.42, *p* = .036, *d* = 0.39.

A closer inspection of the figure hints at a different pattern of effects for the lower-middle class students than the more stable performance of either the middle and upper-middle class students. Up until Quiz 7, lower SES students’ performance started low and continued to drop. Thereafter, however, their performance improved until the last 2-3 weeks of the semester. One possibility is that this drop in performance at the end of the semester may have been the result of students studying harder for their other courses.

**References**

1. Tausczik YR, Pennebaker JW (2013) Improving teamwork using real-time language feedback. CHI ACM 978-1-4503-1899-0/13/04.
